# Natural transmission of feline immunodeficiency virus from infected queen to kitten

**DOI:** 10.1186/1743-422X-9-99

**Published:** 2012-05-25

**Authors:** Sheila de Oliveira Medeiros, Angelica Nascimento Martins, Carlos Gabriel Almeida Dias, Amilcar Tanuri, Rodrigo de Moraes Brindeiro

**Affiliations:** 1 Laboratório de Virologia Molecular Animal, Universidade Federal do Rio de Janeiro, Rio de Janeiro, Brazil; 2 Virus-Cell Interaction Section, HIV Drug Resistance Program, National Cancer Institute at Frederick, Frederick, MD, USA; 3 Laboratório de Virologia Molecular, Departamento de Genética, Universidade Federal do Rio de Janeiro, CCS – Bloco A2, sala 121 – Cidade Universitária – Ilha do Fundão, 21944-970, Rio de Janeiro - RJ, Brazil

**Keywords:** Vertical transmission, FIV, Stray cats

## Abstract

**Background:**

Feline immunodeficiency virus (FIV) is a naturally occurring lentivirus that infects cats. The primary mode of transmission occurs through bite wounds, and other routes are difficult to observe in nature.

**Findings:**

The purpose of this study was to evaluate FIV transmission from queen to kitten in a colony of naturally infected stray cats. With this aim, a queen was monitored over a period of three years. A blood sample was taken to amplify and sequence *gag*, *pol* and *env* regions of the virus from the queen, two kittens and other cats from the colony.

**Conclusion:**

Phylogenetic analysis showed evidence of queen to kitten transmission.

## Findings

Feline immunodeficiency virus (FIV) is a member of the retrovirus group which includes human immunodeficiency virus (HIV) and can cause acquired immune deficiency syndrome in cats [1]. FIV is transmitted mainly by biting [2], which frequently occurs during fights or coitus, as the male bites the female at the neck to restrain her and to control positioning of both animals’ hindquarters [3]. Vertical and sexual transmission is unusual in nature [4,5], but experimental infection of cats with specific strains produces high rates of fetal infection and reproductive failure [6-8].

The average litter size for healthy cats maintained under equatorial natural photoperiod is about 4.82 ± 1.24 kittens (Dias C.G.A., unpublished observations), and may be influenced by many factors, including development of systemic diseases [9]. Data obtained from experimental studies shows that FIV can be transmitted to 70% of the kittens with acute infection of the mothers [10], and the transmission can occur via placenta, during the birth process or through nursing [8,11]. Intrauterine transmission leads to several pathogenic consequences including arrested fetal development, abortion, stillbirth, subnormal birth weight, and birth of viable, virus-infected and T-cell-deficient but asymptomatic kittens [8,12]. In nature, FIV-positive queens rarely infect their offspring. This is believed to result from the biological characteristics of the virus, in which a high viral burden is produced for only a few weeks after infection. After the acute stage of infection, the plasma antibody titer rises, circulating viral antigens become undetectable, and vertical transmission becomes unlikely [13]. Carpenter *et al.* first demonstrated natural vertical transmission in pumas in 1996 [14]. Herein we present indirect evidence of natural transmission of FIV from queen to kitten in a group of stray domestic cats living in an urban environment.

The data were obtained from a colony of 20 cats living on private property in the northern district of Rio de Janeiro, Brazil. The cohort was comprised mainly of stray cats, although owned cats that roam freely sometimes appeared. The colony was visually observed for five years. During this period, the number of cats dropped from twenty to one untamed female cat (queen). This queen was monitored for three years. She was very aggressive and she was constantly isolated. During the breeding season two tomcats were seen roaming in the area. The queen had two litters per year and she aborted twice. The average number of neonates per litter was 1 to 2. Eleven cats from the colony, including the queen (RJ48), two tom cats (RJ34 and RJ35) and three kittens - two from one litter (RJ51 and RJ52) and one from the other litter (RJ36) - were captured in cage traps or restrained manually. The age of the kittens when captured varied between 6 to 8 weeks, because the queen rejected them after weaning. All captured cats were neutered and adopted. A blood sample (3 mL) was collected by venipuncture (jugular or femoral) and placed in a tube with anticoagulant (EDTA). Peripheral blood mononuclear cells (PBMC) were separated from plasma by centrifugation. Genomic DNA was extracted and FIV provirus was amplified by nested PCR as described previously [15], using primers directed to FIV *gag* (CA), *pol* (RT) and *env* (V3-V4) regions, generating fragments with 405 bp, 603 bp and 554 bp, respectively. These genomic regions were amplified by PCR from the queen (RJ48), a kitten (RJ36), the two males (RJ34 and RJ35) and another female that was captured (RJ47) (Table 1). Plasma samples of these five cats were examined for FIV antibodies and feline leukemia virus (FeLV) antigens, using the ELISA test SNAP FIV/FeLV Combo Kit (IDEXX Laboratories) according to manufacturer’s instructions, with positive results to FIV antibodies and negative results to FeLV antigens (Table 1). Using the ClustalW tool [16], sequences from each region were aligned with reference sequences representing closely related viruses that were obtained from GenBank (http://www.ncbi.nlm.nih.gov/nucleotide) and from our previous study [15]. The phylogenetic analyses were performed by the neighbor-joining method with MEGA 4 software [17]. The distance matrix and the phylogenetic trees were generated using Kimura's two-parameter model for nucleotides.

**Table 1:** Characterization of samples in this study

**Animal**	**Sex**	**Serological status**		**PCR** ***gag, RT, env***
		**FIV**	**FeLV**	
RJ34 (tomcat 1)	M	+	-	+
RJ35 (tomcat 2)	M	+	-	+
RJ36 (kitten)	F	+	-	+
RJ47	F	+	-	+
RJ48 (queen)	F	+	-	+
RJ50	F	ND	ND	-
RJ51 (kitten)	F	ND	ND	-
RJ52 (kitten)	F	ND	ND	-
RJ58	F	ND	ND	-
RJ59	F	ND	ND	-
RJ60	F	ND	ND	-

ND – not determined.

**Figure 1:**
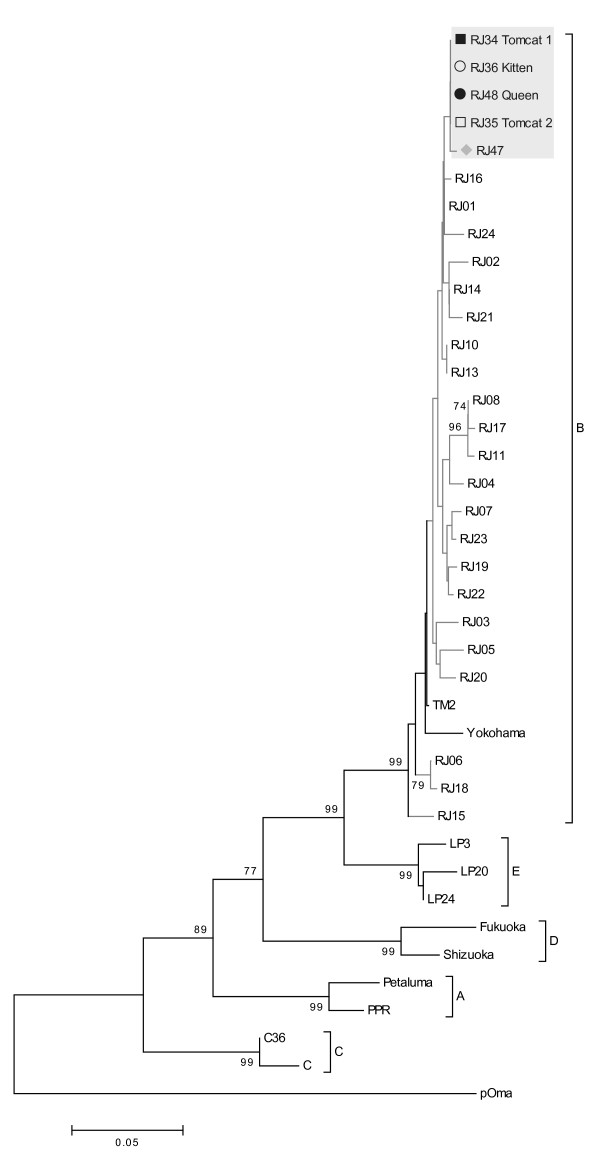
**Neighbour-joining phylogenetic tree based on alignment of 362 nucleotides (nt) from CA-*gag *region.** Bootstrap values based on 1000 replicates. Values greater than 70% are shown. Brazilian samples are represented by grey branch lines, and samples from the study are represented by a gray box and symbols: the black circle represents sequences from the queen (RJ48); the white circle, those from the kitten (RJ36); the gray lozenge, those from the female cat (RJ47); the black square, those from tomcat 1 (RJ34); and the white square, those from tomcat 2 (RJ35). The horizontal bar indicates the nucleotide substitution scale. Reference sequences from GenBank are: Subtype A - From USA: Petaluma (M25381), PPR (M36968). Subtype B - From Japan: TM2 (M59418), Yokohama (D37819). From USA: USIL2489 (U11820). From Brazil: RJ01 to RJ08 (EU375620 to EU375627); RJ10 and RJ11 (EU375629 and EU375630); RJ13 to RJ24 (EU375632 to EU375643); RJ34 and RJ35 (EU375644 and EU375645). Subtype C – From USA: C36 (AY600517). From Canada: C (AF474246). Subtype E – From Argentina: LP3 (AB027302), LP20 (AB027303), LP24 (AB027304). Subtype D – From Japan: Fukuoka (D37822), Shizuoka (D37818). Selvage isolate from Pallas cat (AY713445).

**Figure 2:**
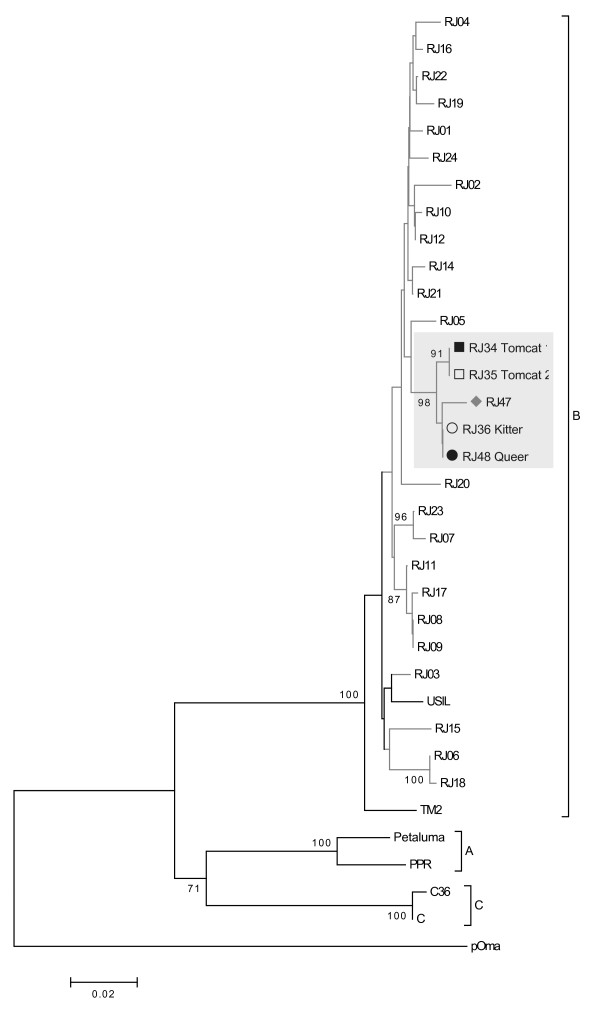
**Phylogenetic tree from 554 nt of RT-*pol *alignment.** Tree was inferred as described in Figure 1. Symbols used in this figure are the same as those used in Figure 1. Reference sequences from GenBank are: Subtype A - From USA: Petaluma (M25381), PPR (M36968). Subtype B - From Japan: TM2 (M59418). From USA: USIL2489 (U11820). From Brazil: RJ01 to RJ08 (EU375568 to EU375575); RJ10 and RJ11 (EU375577 and EU375578); RJ13 to RJ24 (EU375580 to EU375591); RJ34 and RJ35 (EU375592 and EU375593). Subtype C – From USA: C36 (AY600517). From Canada: C (AF474246). Selvage isolate from Pallas cat (AY713445).

**Figure 3:**
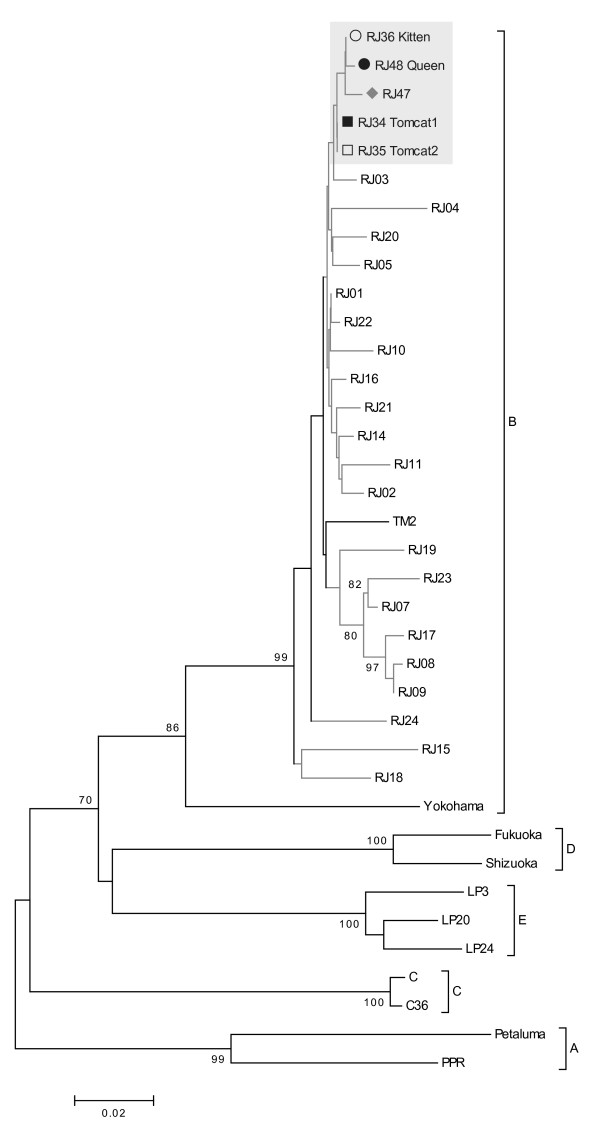
**Neighbour-joining phylogenetic tree based on alignment of 487 nt from V3-V4-*env *region.** Reference sequences from GenBank are: Subtype A - From USA: Petaluma (M25381), PPR (M36968). Subtype B - From Japan: TM2 (M59418), Yokohama (D37812). From Brazil: RJ01 to RJ11 (EU375594 to EU375604); RJ14 to RJ24 (EU375607 to EU375617); RJ34 and RJ35 (EU375618 and EU375619). Subtype C – From USA: C36 (AY600517). From Canada: C (AF474246). Subtype D: From Japan: Fukuoka (D37815), Shizuoka (D37811). Subtype E: From Argentina: LP3 (D84496), LP20 (D84498), LP24 (D84500).

The phylogenetic trees were constructed based on majority viral populations, and were similar for the *gag* (CA), *pol* (RT) and *env* (V3-V4) regions. We previously showed that only subtype B was circulating in samples of infected domestic cats from the city of Rio de Janeiro [15]. Sequences from these cats grouped with subtype B isolates and displayed a monophyletic clade, distinct from other sequences from Rio de Janeiro (Figures 1,2 and 3). Sequences from the two tomcats (RJ34 and RJ35) grouped together in the three genomic regions (Figures 1,2 and 3). To confirm the sequence profile and negate contamination problems, viral RNA (vRNA) was extracted from plasma samples using the QIAamp viral RNA Mini Kit (QIAGEN, Germany) and were sequenced, as the cats were donated and taken to another city in Rio de Janeiro State and a new blood sample analysis could not be performed.

The only distance found between the isolates from queen and kitten was in *env* gene (Table 2), and could be explained by natural selection during transmission, a completely different bottleneck compared with the main routes of FIV transmission. Sequences from a non-related FIV-infected cat from the same geographic area (RJ47) were evaluated to estimate what would be a regular genetic distance between non-related virus circulating and were segregated in different branches of the trees (Figures 1, 2 and 3). This suggests that epidemiologically linked sequences were evolutionarily closer to each other than to unlinked sequences.

**Table 2:** Pairwise distance between samples

***gag *CA**	**RJ34 Tomcat 1**	**RJ35 Tomcat 2**	**RJ36 Kitten**	**RJ47**
**RJ34 Tomcat 1**				
**RJ35 Tomcat 2**	0.0%			
**RJ36 Kitten**	0.0%	0.0%		
**RJ47**	0.3%	0.3%	0.3%	
**RJ48 Queen**	0.0%	0.0%	0.0%	0.3%
***pol *RT**				
**RJ34 Tomcat 1**				
**RJ35 Tomcat 2**	0.0%			
**RJ36 Kitten**	0.5%	0.5%		
**RJ47**	1.3%	1.3%	0.7%	
**RJ48 Queen**	0.5%	0.5%	0%	0.7%
***env *V3-V4**				
**RJ34 Tomcat 1**				
**RJ35 Tomcat 2**	0.0%			
**RJ36 Kitten**	0.4%	0.4%		
**RJ47**	1.0%	1.0%	0.8%	
**RJ48 Queen**	0.6%	0.6%	0.2%	1.0%

This sequence data provided indirect evidence for queen to kitten transmission in naturally infected domestic cats, and indicate that the two males were infected with the same strain. Our hypothesis is that, as the animals were removed from the original group, the reduced number of males led to an increase in the occurrence of fights over females, thus facilitating the transmission of FIV between them.

Mother to offspring FIV transmission was not observed in a nine-month period of study (from June 1990 until March 1991) in a closed breeding colony composed of 22 females and 3 males [18]. This study could not recover FIV in cell culture from kitten PBMC. As has been described, cats exhibit translocation of kitten behavior around the third week after partum. This behavior occurs when the queen is under stress-inducing circumstances not normally observed in experimentally controlled environments, and needs to be investigated with regard to the potentiality for FIV transmission. Another maternal behavior directed at kittens could be involved, like umbilical cord chewing and anogenital licking, as well as other behaviors during the nursing period [9]. O’Neil *et al.* (1996) have reported a smaller litter size, abortion and fetal resorption in females experimentally infected with FIV, which is compatible with our observation of two abortions and a small litter size [7]. These data suggest deleterious effects of maternal FIV infection on reproductive parameters in this female.

## Competing interests

The authors declare that they have no competing interests.

## Authors’ contributions

SOM wrote the manuscript and carried out the molecular studies. ANM participated in the sequence alignment and the phylogenetic analysis. CGAD contributed to data analysis. RMB participated in the design of the study. AT participated in the design and coordination of the study. All authors edited and approved the final manuscript.

## Authors’ information

^1^Laboratório de Virologia Molecular Animal, Universidade Federal do Rio de Janeiro, Rio de Janeiro, Brazil. ^2^Virus-Cell Interaction Section, HIV Drug Resistance Program, National Cancer Institute at Frederick, Frederick, MD, USA.
